# Detection of *Pasteurella multocida* and macrolide resistance genes in calf pneumonia: an immunohistochemical and molecular approach

**DOI:** 10.1007/s11250-026-05119-1

**Published:** 2026-06-08

**Authors:** Mehmet Burak Ates, Mustafa Ortatatli, Funda Terzi, Asli Balevi, Zafer Sayin, Ozgur Ozdemir, Fatih Hatipoglu

**Affiliations:** 1 Department of Pathology, Faculty of Veterinary Medicine, Selçuk University, Konya, TR-42130 Türkiye; 2 Department of Pathology, Faculty of Veterinary Medicine, Kastamonu University, Kastamonu, TR- 37150 Türkiye; 3 Department of Microbiology, Faculty of Veterinary Medicine, Selçuk University, Konya, TR-42130 Türkiye

**Keywords:** Bovine respiratory disease, Calf mortality, Antibiotic, FFPE tissue

## Abstract

This study aimed to detect *Pasteurella multocida* by immunohistochemical (IHC) methods in formalin-fixed paraffin-embedded (FFPE) lung tissues obtained from pneumonic calves and to investigate the presence of macrolide resistance genes (*erm(42)*,* msr(E)*,* mph(E)*) in positive cases using real-time PCR. Among lung tissues collected from 100 necropsied calves, histopathological analysis revealed fibrinous bronchopneumonia as the most common diagnosis (56/100). *P. multocida* was detected immunohistochemically in 55 out of 100 cases (55%) using a specifically produced polyclonal antibody. The highest prevalence of *P. multocida* was observed in cases of fibrinous bronchopneumonia (82%). At least one of the targeted macrolide resistance genes was detected in 39 (71%) of the 55 IHC-positive cases. The most frequently encountered resistance profiles were the *msr(E)* + *mph(E)* combination (*n* = 12; 22%) and the triple gene combination *erm(42) + msr(E) + mph(E)* (*n* = 12; 22%). In conclusion, this study demonstrated that *P. multocida* plays a key role in fibrinous pneumonia in calves and that these strains harbor high levels and diverse types of macrolide resistance genes, which may contribute to treatment failures. Therefore, routine molecular resistance screening and the development of rational therapeutic strategies are recommended for veterinary practice.

## Introduction

Bovine Respiratory Disease (BRD) complex is one of the major causes of mortality and morbidity in calves and leads to significant economic losses worldwide (Blakebrough-Hall et al. 2020). Respiratory tract infections spread rapidly, particularly in intensive and confined calf-rearing systems, and the disease typically has a multifactorial etiology. Among the bacterial agents involved in this complex, *Pasteurella multocida* is a major cause of acute fibrinous bronchopneumonia. This microorganism, which normally resides as a facultative pathogen on the upper respiratory mucosa, can migrate to the lower respiratory tract under the influence of stress factors (transportation, dietary changes, climatic conditions, viral infections, etc.) and cause severe pulmonary lesions (Caswell and Williams 2016).

*P. multocida* possesses various virulence factors, including a capsule, outer membrane proteins, iron-binding proteins, adhesins, neuraminidase, and lipopolysaccharides (Boyce and Adler 2000). Among these, serotype A:3 is one of the most frequently isolated variants causing pneumonia in cattle (Harper et al. 2006). Clinically, infections are characterized by symptoms such as high fever, depression, coughing, and mucopurulent nasal discharge. Pathologically, they manifest as fibrinous bronchopneumonia with necrotic and exudative lesions, particularly in the cranioventral lung lobes (Panciera and Confer 2010).

Macrolide antibiotics are among the most commonly used antimicrobials in BRD cases. Agents such as tilmicosin, tulathromycin, tildipirosin, and gamithromycin are widely preferred due to their broad-spectrum activity; however, increased usage has led to the emergence of resistance. Recent studies have shown that specific genetic elements play a key role in macrolide resistance. In particular, the genes *erm(42)*,* msr(E)*, and *mph(E)*, in various combinations, have been shown to contribute to resistance and significantly increase minimum inhibitory concentrations (MIC) (Desmolaize et al. 2011b; Woolums et al. 2018).

It is well established that traditional culture and antibiogram methods may fail to detect pathogens, especially in animals that have undergone antimicrobial treatment or in carcasses that are autolyzed due to delayed necropsy. This limitation may result in missed diagnoses and incorrect antibiotic selection. Therefore, immunohistochemical analyses and PCR-based molecular tests conducted on formalin-fixed paraffin-embedded tissues offer more reliable and practical diagnostic alternatives (Schmitt et al. 2013).

The recent increase in cases of calf mortality due to pneumonia that do not respond to treatment suggests that antibiotic resistance has become a serious issue in the field (Vinayamohan et al. 2022). The widespread and sometimes irrational use of macrolides has contributed to the emergence of resistant strains of respiratory pathogens such as *P. multocida*. This not only leads to treatment failures but also exacerbates economic losses at the herd level. Considering the limitations of current culture and antibiogram methods, the adoption of more sensitive and rapid molecular techniques in veterinary field diagnostics is of great importance. The aim of this study was to detect *P. multocida* in pneumonic calves using immunohistochemistry and to investigate the presence of macrolide resistance genes (*erm(42)*,* msr(E)*,* mph(E)*) in IHC-positive cases using real-time PCR. The ultimate goal is to contribute to the scientific reorganization of antibiotic treatment protocols and to develop effective diagnosis and control strategies to reduce calf losses due to pneumonia.

## Materials and methods

### Animals, study design, ethical status

The study material consisted of lung tissues from 100 calves submitted to the Department of Pathology at Selçuk University Faculty of Veterinary Medicine for necropsy between 2012 and 2017, with a clinical and gross diagnosis of pneumonia. During systematic necropsy, lung samples were collected following macroscopic evaluation and prepared for histopathological and molecular analyses. The study protocol was approved by the Experimental Animal Production and Research Ethics Committee of Selçuk University Faculty of Veterinary Medicine (Approval no: 2017/63).

### Histopathological method

Lung tissues obtained from necropsied calves were fixed in 10% neutral buffered formalin solution for at least 48 h. After fixation, tissue samples were processed through routine histological procedures and embedded in paraffin blocks. Sections of 5 μm thickness were cut using a microtome (Leica RM2125RT, Germany) and placed on adhesive slides. The slides were stained with hematoxylin and eosin (H&E) following standard protocols. After staining, slides were mounted with Entellan and examined under a light microscope (Olympus BX51, Tokyo, Japan). Each sample was morphologically classified based on the form of pneumonia and characteristic histopathological findings were documented photographically (Olympus DP12). Based on findings such as exudates in alveoli, cellular debris in bronchiolar lumens, septal thickening, necrotic foci, and “oat cell” formations, the samples were categorized into fibrinous, fibrinopurulent, catarrhal, suppurative-abscess-forming, interstitial, and other types (aspiration, granulomatous) of bronchopneumonia.

### Immunohistochemical method

For immunohistochemical (IHC) analysis, 5 μm thick lung tissue sections obtained from paraffin blocks were placed on adhesive slides and processed using a fully automated staining system (Leica Bond-Max, Germany). The Bond™ Polymer Refine Detection Kit (Leica, DS9800) was used, and the procedure followed the kit’s protocol, including steps such as peroxidase block, protein block, post-primer, polymer, DAB, and counterstaining with hematoxylin. Antigen retrieval was performed with Epitope 1 (citrate buffer, pH: 6.0) at 100 °C for 30 min. Samples were incubated at room temperature for 45 min with an anti-*P. multocida* primary antibody (1:1500). Stained sections were examined under a light microscope (Olympus BX51), and structures showing positive immunoreactivity were photographed using a digital camera system (Olympus DP12). Immunoreactions were evaluated based on the presence of brown DAB deposits in the cytoplasm of neutrophils, macrophages, and epithelial cells in bronchi, bronchioles, and alveolar lumens. The degree and distribution of positivity were analyzed in comparison with the pneumonia types.

### *Production of Pasteurella multocida polyclonal antibody for immunohistochemical staining *

**Fig. 1:**
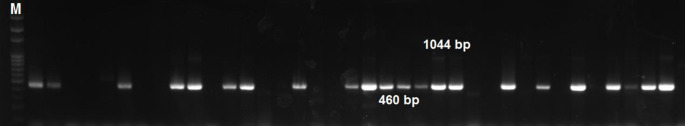
Agarose gel image of *Pasteurella multoicida* confirmed by PCR. M: Marker, 460 bp: Pasteurella multocida; 1044 bp: Capsular antigen A positive isolates

The *Pasteurella multocida* isolate used for antibody production was obtained from the bacterial culture collection of the Department of Microbiology. To produce the primary antibody for IHC staining, the identity of the *P. multocida* strain was first confirmed and a hyperimmunization process was carried out. DNA was extracted from bacterial isolates using the Wizard^®^ Genomic DNA Purification Kit (Promega, USA), and multiplex PCR (mPCR) was performed using KMT1 primers for species-level identification of *P. multocida* (KMT1F 5’-ATCCGCTATTTACCCAGTGG-3’, KMT1R 5’-GCTGTAAACGAACTCGCCAC-3’) and capA primers for capsule antigen A (capAF 5’-TGCCAAAATCGCAGTCAG-3’, capAR 5’-TTGCCATCATTGTCAGTG-3’) (Townsend et al. 1998). PCR products were visualized under UV light using agarose gel electrophoresis (Fig. 1).

The confirmed *P. multocida* isolate was inactivated with 3% formalin to prepare the antigen. After sterilization controls, a bacterial suspension equivalent to McFarland standard No. 4 (~ 2 × 10⁹ CFU/mL) was obtained. The inactivated culture was prepared in two forms: one without adjuvant (antigen) and the other with Montanide ISA 206 VG (Seppic, France) as adjuvant (vaccine). These preparations were stored at 4 °C for future use.

Four Swiss albino mice were used for the production of hyperimmune serum. Each mouse received 0.1 mL of antigen without adjuvant intramuscularly every 5 days for 30 days. On day 30, 0.2 mL of the adjuvanted vaccine was administered, followed by a final booster dose of 0.2 mL on day 45. On day 60, blood was collected via cardiac puncture. Sera were separated by centrifugation and stored at − 20 °C (Chung et al. 2005). The resulting hyperimmune serum was used as the primary antibody in IHC staining, and its specificity against *P. multocida* antigens was experimentally confirmed.

### Real-time PCR method and primer design

In cases found to be immunohistochemically positive for *Pasteurella multocida*, real-time PCR was used to evaluate the development of genetic resistance to macrolide antibiotics. This analysis was performed on DNA extracted from lung tissues embedded in paraffin blocks. DNA isolation was carried out from formalin-fixed, paraffin-embedded (FFPE) tissue samples using the QIAamp DNA FFPE Tissue Kit (Qiagen, Cat. No: 56404) according to the manufacturer’s protocol. Tissue sections (three 5-µm thick slices) were subjected to deparaffinization and rehydration through xylene and alcohol series, followed by proteinase K digestion for cell lysis, and then a column-based purification procedure. The isolated DNA samples were stored at − 20 °C until further use.

Real-time PCR amplifications were conducted on a Roche LightCycler 96 system using hydrolysis probe (TaqMan) technology. Reactions were prepared in a total volume of 20 µL, consisting of 10 µL Master Mix, 1 µL forward primer, 1 µL reverse primer, 1 µL fluorescently labeled probe, 2 µL PCR-grade distilled water, and 5 µL DNA template. The thermal cycling protocol included an initial denaturation (pre-incubation) at 95 °C for 10 min, followed by 45 cycles of 95 °C for 15 s (denaturation) and 64 °C for 45 s (annealing). A final cooling step was performed at 37 °C for 30 s.

The primer sequences for macrolide resistance genes used in this study were defined by Rose et al. (2012) and are listed below (Table 1):

**Table 1:** Primer sequences, amplicon sizes, and GenBank accession numbers used for the detection of macrolide resistance genes by real-time PCR

Gen	Primary Sequence (5’ – 3’)	Fragment size (bp)	GenBank No
*erm(42)*	F: TGCACCATCTTACAAGGAGT R: CATGCCTGTCTTCAAGGTTT	173	HQ888763
*mph(E)*	F: ATGCCCAGCATATAAATCGC R: ATATGGACAAAGATAGCCCG	271	JF769133
*msr(E)*	F: TATAGCGACTTTAGCGCCAA R: GCCGTAGAATATGAGCTGAT	395	JF769133

Separate reactions labelled with fluorescent probes were set up for each gene, and each sample was run in technical triplicates. Samples with a cycle threshold (Ct) value below 35 were considered positive.

## Results

### Macroscopic findings

Macroscopic examination of lung tissues from 100 necropsied calves revealed distinct lesions reflecting various forms of bronchopneumonia. In most cases, red-brown consolidated (hepatized) areas were distributed in both cranial and caudal lobes, accompanied by marked expansion of interlobular septa, yellow-white fibrinous exudate on the pleural surface, and pleural adhesions. In some specimens, opaque, mucopurulent, or purulent exudate was seen oozing from the cut surfaces. In several cases, abscess formations of varying size containing purulent material were detected, while in others, the lungs appeared spongy and elastic with visible rib impressions, showing no signs of collapse.

In fibrinous bronchopneumonia cases, the lobular margins were distinctly demarcated, the pleural surfaces appeared dull and covered with fibrin, and cut surfaces showed abundant fibrinous exudation. In catarrhal forms, irregular red areas and seromucous exudate were observed. Suppurative and abscess-forming cases showed localized, thickly encapsulated foci of yellow-white, viscous pus within the lung tissue. In interstitial pneumonia, the absence of lobar collapse, elastic consistency, smooth surface texture, and the presence of nodular lesions and pleural effusion were noted.

### Histopathologic findings

**Fig. 2:**
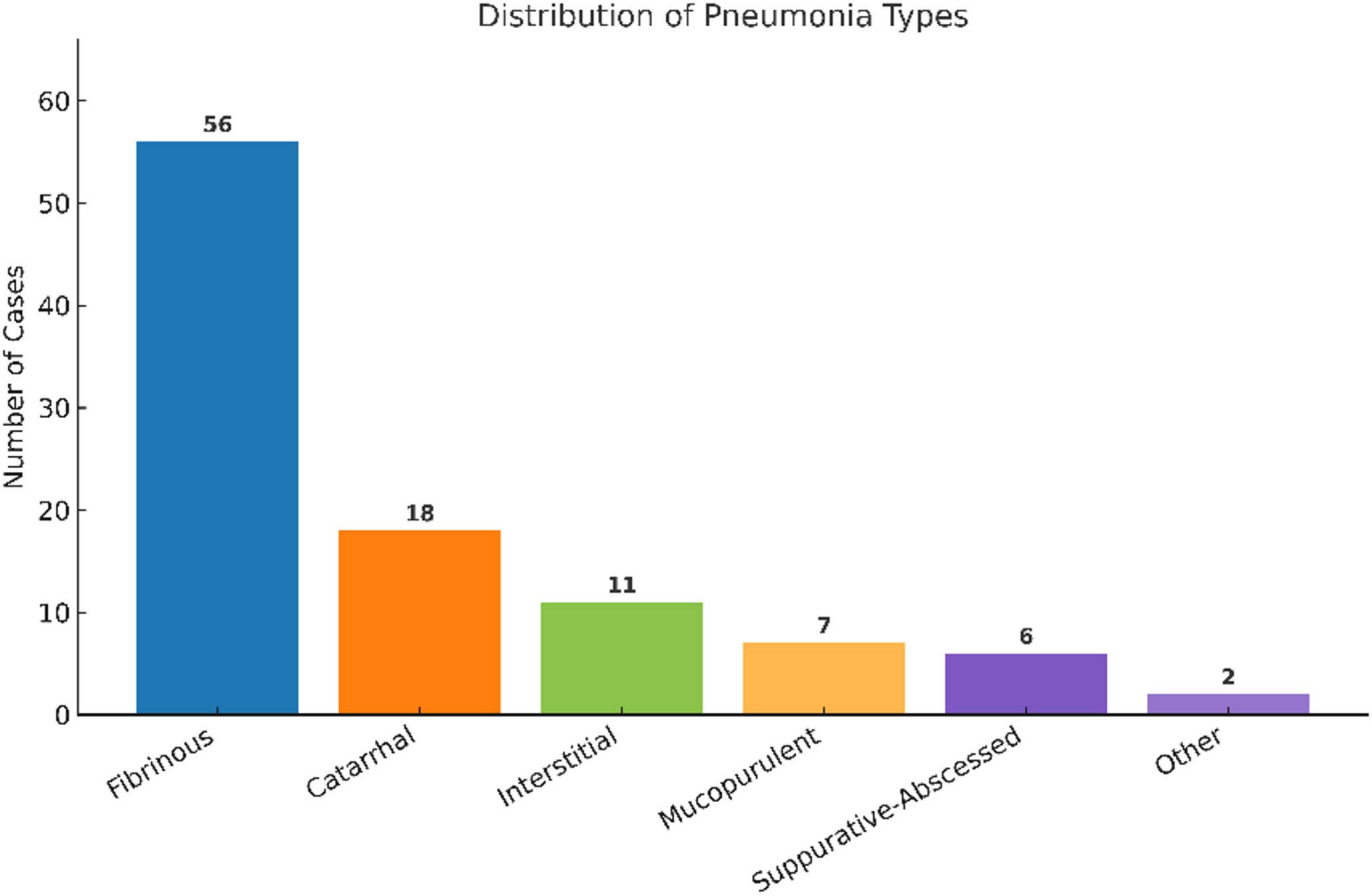
Distribution of pneumonia types in calves

Histopathological examination classified the lung samples from 100 calves into various types of pneumonia and evaluated their microscopic lesions in detail. Based on this classification, 56 cases were diagnosed with fibrinous bronchopneumonia (fibrinous, fibrinopurulent, fibrinonecrotic), 18 with catarrhal bronchopneumonia, 7 with catarrhal-suppurative bronchopneumonia, 6 with suppurative-abscess-forming pneumonia, 11 with interstitial pneumonia, 1 with aspiration pneumonia, and 1 with granulomatous pneumonia (Fig. 2).

**Fig. 3:**
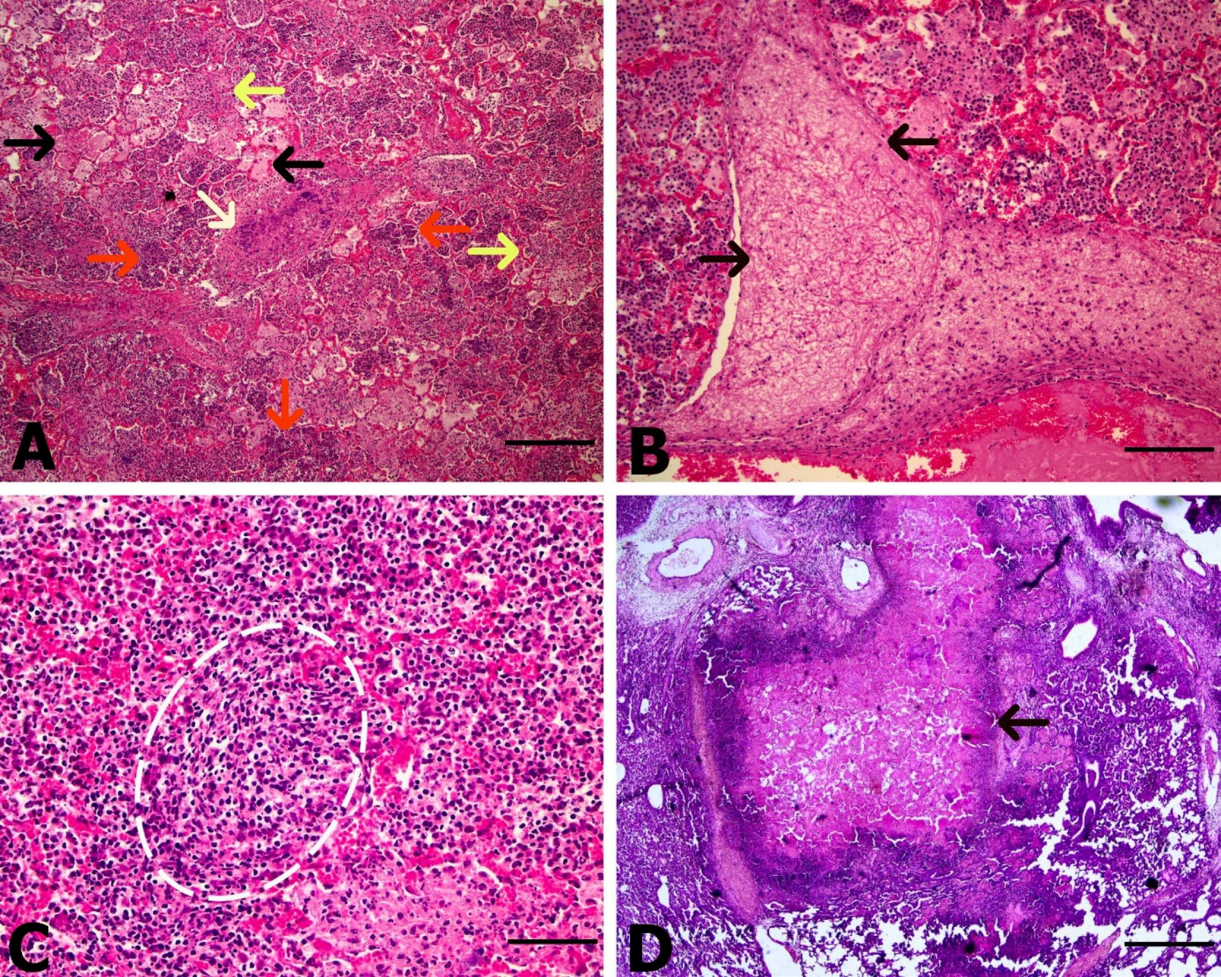
Histopathological micrographs. **A**: Stages of fibrinous pneumonia; inflammatory hyperemia (black arrows), red hepatization (yellow arrows), gray hepatization (red arrows), necrosis and bacterial colonies (white arrow), H&E, Scale bar: 200 μm. **B**: Widening of the interlobular septa due to inflammatory edema and fibrin (black arrows), H&E, Scale bar: 100 μm. **C**: Oat cell formations, H&E, Scale bar: 50 μm. **D**: Suppurative-abscess-forming pneumonia. A large area of liquefactive necrosis (black arrow), H&E, Scale bar: 500 μm

In fibrinous bronchopneumonia, red and gray hepatization phases were noted, characterized by congestion in interalveolar septa and the presence of neutrophils, desquamated epithelial cells, and dense fibrin strands in alveolar spaces (Fig. 3A). Marked thickening of the interlobular septa due to fibrinous exudation was observed (Fig. 3B). Some cases exhibited irregularly shaped necrotic foci surrounded by inflammatory cells and oat cell formations (Fig. 3C).

In catarrhal bronchopneumonia, seromucous exudate accumulation in alveoli, focal emphysema, atelectatic areas, and moderate neutrophilic infiltration were observed. Catarrhal-suppurative cases additionally showed mucopurulent exudate in alveolar and bronchiolar lumens. Suppurative-abscess-forming pneumonias had encapsulated abscesses with intense neutrophil accumulation and necrotic contents within the lung parenchyma (Fig. 3D). Bronchi and bronchioles exhibited mucopurulent plugs, epithelial damage, and cellular debris.

In interstitial pneumonia, mononuclear cell infiltration and fibrosis were observed in interalveolar septa, along with peribronchiolar and perivascular lymphoid hyperplasia and bronchiolitis obliterans. Alveolar spaces were mostly air-filled and lacked marked exudation. The aspiration pneumonia case exhibited foreign particles (likely milk) in bronchiolar lumens, necrotic areas, and intense neutrophilic infiltration. The granulomatous pneumonia case revealed granulomas consisting of epithelioid macrophages, multinucleated giant cells, and lymphocyte infiltration.

### Immunohistochemical findings

**Fig. 4:**
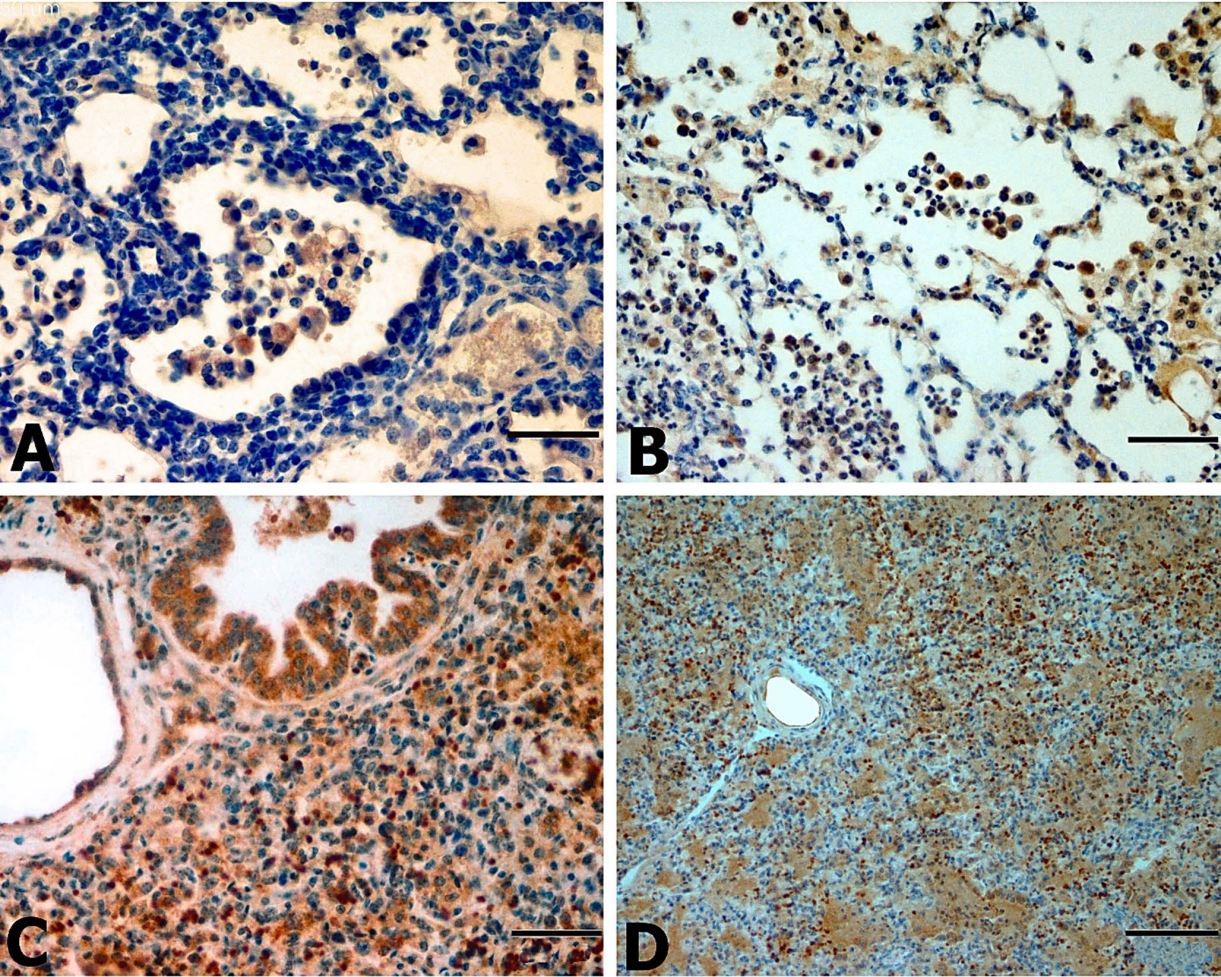
Immunohistochemical microphotographs. **A-B**: *P. multocida* positive immunoreactivity in inflammatory cells, desquamated epithelial cells and inflammatory cells in alveolar lumens, IHC, Scale bar: 50 μm; **C**: *P. multocida* positive immunoreactivity in bronchial epithelial cells and mononuclear cells in interstitial area, IHC, Scale bar: 50 μm, **D**: *P. multocida* positive immunoreactivity in inflammatory cells, desquamated epithelial cells and alveolar macrophages in alveolar lumens, IHC, Scale bar: 200 μm

Immunohistochemical analysis revealed the presence of *Pasteurella multocida* in 55 out of 100 lung samples. Positive immunoreactivity was observed as brown DAB deposits in the cytoplasm of neutrophils, alveolar macrophages, and occasionally epithelial cells in the bronchi, bronchioles, and alveolar lumens (Fig. 4).

**Fig. 5:**
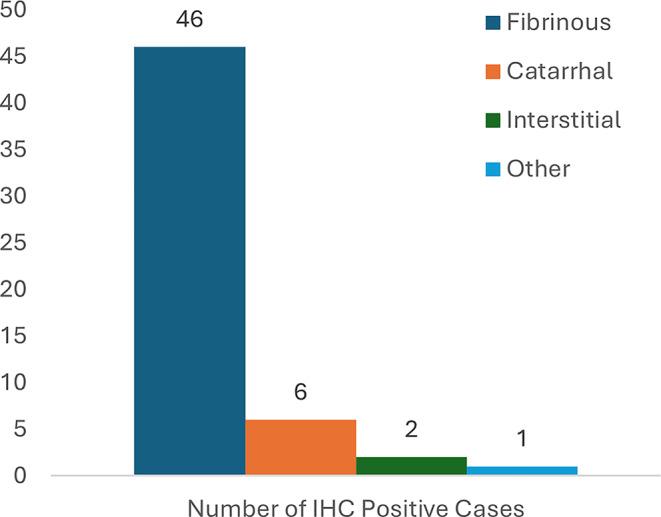
*P. multocida* positive cases according to pneumonia types

When IHC positivity rates were evaluated by pneumonia type, 46 out of 56 fibrinous bronchopneumonia cases (82%), 6 out of 18 catarrhal bronchopneumonia cases (33%), 2 out of 11 interstitial pneumonia cases (18%), and 1 out of 15 cases in the “other” category (aspiration pneumonia) (7%) tested positive for *P. multocida*. No IHC positivity was detected in suppurative-abscess-forming or granulomatous pneumonia cases (Fig. 5).

### Real-time PCR and resistance genes findings

Real-time PCR was used to detect the presence of *erm(42)*, *msr(E)*, and *mph(E)* resistance genes in 55 calves that were IHC-positive for *P. multocida*. At least one resistance gene was detected in 39 of these 55 cases (71%). The *erm(42)* gene alone was detected in only 2 cases (3.6%), *msr(E)* alone in 5 cases (9.1%), and *mph(E)* alone in 7 cases (12.7%). The *msr(E) + mph(E)* combination was found in 12 cases (21.8%), while *mph(E) + erm(42)* was detected in just one case (1.8%). Notably, 12 cases (21.8%) harbored all three resistance genes (*erm(42)*,* msr(E)*,* and mph(E)*). In the remaining 16 cases (29.1%), none of the tested resistance genes were detected.

When the distribution of resistance genes was evaluated according to pneumonia type, the highest positivity rate and genetic diversity were observed in fibrinous bronchopneumonia cases. Of the 46 IHC-positive cases in this group, 35 (76%) had at least one macrolide resistance gene: 5 had *msr(E)* alone, 6 had *mph(E)* alone, 11 had *msr(E) + mph(E)*, 1 had *mph(E) + erm(42)*, and 12 had the triple combination. In 11 cases, no resistance genes were detected (Table 2).

**Table 2:** Distribution of macrolide resistance gene profiles by pneumonia type

	Fibrinous Pneumonia (*n*: 56)	Catarrhal Pneumonia (*n*:18)	Interstitial Pneumonia (*n*:11)	Other (*n*:15)	Total (*n*:100)
*P*. *multocida* (+)(IHC)	46 (%82)	6 (%33)	2 (%18)	1 (%7)	55
*erm (42)*	-	2	-	-	2 (%3.6)
*msr (E)*	5	-	-	-	5 (%9.1)
*mph (E)*	6	-	1	-	7 (%12.7)
*msr (E) + mph (E)*	11	1	-	-	12 (%21.8)
*mph(E) + erm(42)*	1	-			1 (%1.8)
*msr (E) + mph (E) + erm (42)*	12	-	-	-	12 (%21.8)
No expression	11	3	1	1	16 (%29.1)

In the catarrhal bronchopneumonia group, 3 out of 6 IHC-positive cases (50%) carried resistance genes. Of these, 2 had *erm(42)* alone and 1 had *msr(E) + mph(E)*. The remaining 3 cases had no detectable resistance genes. Among the 2 IHC-positive interstitial pneumonia cases, only one showed *mph(E)* positivity.

Of the 15 cases in the “other” category (catarrhal-suppurative, suppurative-abscess-forming, aspiration, and granulomatous pneumonia), only one was IHC-positive for *P. multocida*, but no resistance genes were detected in that case.

## Discussion

Calf pneumonia is one of the leading causes of morbidity and mortality in cattle farming worldwide, resulting in significant economic losses. The aim of this study was to investigate the presence of *Pasteurella multocida* in pneumonic calves and to determine the incidence and distribution of macrolide resistance genes (*erm(42)*,* msr(E)*,* mph(E)*) in positive cases using immunohistochemical and molecular approaches. The findings revealed that *P. multocida* was frequently involved in cases of fibrinous bronchopneumonia, and 71% of these strains carried macrolide resistance genes.

Histopathological evaluation showed that fibrinous bronchopneumonia was the most prevalent diagnosis (56%) among the 100 necropsied calves. Macroscopically, the observations aligned with previous reports in the literature, including red and gray hepatization in the cranial and medial lobes, pleural fibrin accumulation, and pleural adhesions (Schiefer et al. 1978; Booker et al. 2008; Panciera and Confer 2010; López and Martinson 2017; Ciftci et al. 2018). As noted by Caswell and Williams (2016), such lesions can lead to severe respiratory distress, posing a significant risk to the survival of young calves. Moreover, the high IHC positivity for *P. multocida* in fibrinous bronchopneumonia cases (82%) highlights the pathogen’s crucial role in the disease pathogenesis. The role of *P. multocida* as a primary agent in calf pneumonia has been consistently reported in various studies (Haziroglu et al. 1997; Dabo et al. 2007; Elbatawy et al. 2022). Particularly in neonatal or recently weaned calves with low immunity, environmental stressors can markedly increase the risk of bacterial infection (Cho and Yoon 2014; Hulbert and Moisá 2016). These conditions suggest that *P. multocida* can rapidly proliferate and invade alveolar structures when host defences are compromised, playing a major role in the development of severe forms such as fibrinous bronchopneumonia.

Traditionally, bacterial culture has been the main method for identifying bacterial pneumonia agents (Nagaraja 2022). However, in cases where animals have received antibiotic treatment, or when there is a delay in necropsy leading to decomposition, culture results may fail to detect pathogens. Sources indicate that IHC is a reliable alternative in such cases—especially when fresh tissue is unavailable or when antimicrobial therapy or autolysis interferes with pathogen recovery (Horadagoda and Belak 1990; Oliveira Filho et al. 2015). In our study, IHC detection of the pathogen allowed us to overcome the limitations posed by prior antibiotic use, autolysis, and negative culture results, minimizing the risk of underdiagnosis. This finding underscores the utility of IHC as an important diagnostic tool for timely and accurate pathogen identification in field conditions.

The presence of at least one macrolide resistance gene (*erm(42)*,* msr(E)*,* mph(E)*) was detected by real-time PCR in 39 (71%) of 55 cases with immunohistochemically positive *P. multocida*. These findings are consistent with other reports, where one or more resistance genes in *P. multocida* ranged between 60 and 80% (Rose et al. 2012; Desmolaize et al. 2011b). However, a recent study investigating fatal BRD cases reported even higher detection rates, ranging between 76 and 95% (Ates et al. 2025). This disparity suggests that the genetic resistance burden may be more pronounced in severe, non-responsive clinical cases, which significantly contribute to herd mortality.

The most frequent resistance profiles identified were the *msr(E) + mph(E)* combination (*n* = 12) and the triple combination *erm(42) + msr(E) + mph(E)* (*n* = 12), which have also been commonly reported in previous studies (Rose et al. 2012; Michael et al. 2012a; Beker et al. 2018; Alhamami et al. 2021; Dutta et al. 2021; Ates et al. 2025). Macrolides are among the most widely used antibiotics for the treatment of BRD due to their long duration of action and good tissue penetration (de Jong et al. 2014). However, increasing resistance among *P. multocida* isolates has been linked to these genes (Hatipoglu et al. 2024; Rose et al. 2012; Desmolaize et al. 2011b). Additionally, in IHC-positive cases where no resistance gene was detected by PCR (29%), the presence of mutational alterations in the 23S rRNA gene, as reported in the literature, or other resistance genes not screened in this study is likely (Olsen et al. 2015; DeDonder et al. 2016).

These genes are known to mediate resistance through different mechanisms: *msr(E)* and *mph(E)* act synergistically by promoting antibiotic efflux and chemical modification, respectively, while *erm(42)* encodes a methyltransferase that alters the ribosomal target, resulting in high-level resistance (Rose et al. 2012; Desmolaize et al. 2011a; Leclercq 2002). Notably, strains harbouring multiple resistance genes may display a broad and potent phenotype of antibiotic resistance, contributing to treatment failures (Desmolaize et al. 2011a; Michael et al. 2012a; Rose et al. 2012). Therefore, the findings obtained in this study provide strong evidence supporting the genetic basis of macrolide treatment failures observed in the field, in line with the literature.

The detection of *P. multocida* strains with the triple gene combination at a significant rate of 22% in the current study is also considered concerning. This suggests that not only are these strains capable of inactivating macrolide antibiotics through the mechanisms mentioned above, but also that resistance can spread among different strains in the field. As a matter of fact, it is reported that the ICE (Integrative and Conjugative Elements) structures in which these genes are carried allow horizontal gene transfer and can spread rapidly under field conditions (Beker et al. 2018; Michael et al. 2012b). This finding suggests that the field efficacy of macrolides, one of the most widely used treatment options, may be gradually decreasing. In particular, empirical macrolide treatment strategies may no longer be sustainable in calves highly susceptible to BRD. Indeed, combinations of macrolide resistance genes, particularly the triple profile, have been shown to increase macrolide MIC values by up to 1024-fold, rendering standard therapeutic doses ineffective (Desmolaize et al. 2011a; Michael et al. 2012a; Rose et al. 2012). In this context, the high prevalence of such resistance profiles detected in the present study suggests that macrolide resistance gene burden may represent an important molecular indicator of treatment failure and severe pneumonia outcomes in calves. Treatment-resistant infections are among the primary factors contributing to the mortality rate of pneumonia, the second leading cause of death in calves (Portis et al. 2012). This resistance gene load, combined with the synergistic effects of multiple bacterial pathogens, leads to prolonged inflammation and widespread vascular damage in the lung parenchyma, which may ultimately contribute to increased mortality (Dag et al. 2018; Hatipoglu et al. 2024; Ates et al. 2025). Therefore, it should be noted that persistent or recurrent pneumonia cases, despite treatment, may often be related not only to the presence of the bacterial agent but also to its resistance profile.

Although this study focuses on commonly observed macrolide resistance genes, other antimicrobial resistance markers reported in the literature may also contribute to treatment failure under field conditions. In *P. multocida* isolates, resistance genes such as tetracycline resistance *tet(H)*, β-lactam resistance *blaOXA-2*, and aminoglycoside resistance markers *ant(2’’)-Ia* and *aph(3’’)-Ia* have been identified alongside macrolide resistance genes (Michael et al. 2012a; Beker et al. 2018; Alhamami et al. 2021). In addition, other macrolide resistance markers such as *erm(A)*, *erm(B)*, and *erm(F)*, and mutations affecting resistance-associated regions, may further influence resistance phenotypes and prevalence rates in field strains Li et al. 2016). Many of these genes are carried on ICEs such as *ICEPmu1*, facilitating the horizontal spread of multidrug resistance among respiratory pathogens (Michael et al. 2012b; Beker et al. 2018). Therefore, the resistance profiles identified in this study may represent a portion of a broader reservoir of antimicrobial resistance circulating among *P. multocida* strains associated with calf pneumonia.

## Conclusions

This study highlights the prominent role of *Pasteurella multocida* in the etiology of respiratory infections in calves, especially in fibrinous bronchopneumonia. The combined application of immunohistochemistry and real-time PCR on formalin-fixed paraffin-embedded tissues offers a practical and reliable diagnostic alternative when classical culture techniques fall short. Additionally, the high prevalence of macrolide resistance genes in *P. multocida* strains indicates a significant resistance burden that must be considered in treatment planning. Routine molecular resistance surveillance and the development of targeted therapeutic strategies are essential for reducing pneumonia-associated calf mortality and improving treatment outcomes in veterinary practice.

## Data Availability

The datasets generated during the current study are available from the corresponding author on reasonable request.
